# Segmentation of Brain Tumors Using a Multi-Modal Segment Anything Model (MSAM) with Missing Modality Adaptation

**DOI:** 10.3390/bioengineering12080871

**Published:** 2025-08-12

**Authors:** Jiezhen Xing, Jicong Zhang

**Affiliations:** 1 School of Biological Science and Medical Engineering, Beihang University, Beijing 100191, China; xingjiezhen@buaa.edu.cn; 2 Hefei Innovation Research Institute, Beihang University, Hefei 230012, China

**Keywords:** brain tumor segmentation, multi-modal, MRI, segment anything model, feature fusion

## Abstract

This paper presents a novel multi-modal segment anything model (MSAM) for glioma tumor segmentation using structural MRI images and diffusion tensor imaging data. We designed an effective multimodal feature fusion block to effectively integrate features from different modalities of data, thereby improving the accuracy of brain tumor segmentation. We have designed an effective missing modality training method to address the issue of missing modalities in actual clinical scenarios. To evaluate the effectiveness of MSAM, a series of experiments were conducted comparing its performance with U-Net across various modality combinations. The results demonstrate that MSAM consistently outperforms U-Net in terms of both Dice Similarity Coefficient and 95% Hausdorff Distance, particularly when structural modality data are used alone. Through feature visualization and the use of missing modality training, we show that MSAM can effectively adapt to missing data, providing robust segmentation even when key modalities are absent. Additionally, segmentation accuracy is influenced by tumor region size, with smaller regions presenting more challenges. These findings underscore the potential of MSAM in clinical applications where incomplete data or varying tumor sizes are prevalent.

## 1. Introduction

Brain tumor segmentation is clinically indispensable for treatment planning and research in neuro-oncology. Following radiological diagnosis, accurate tumor segmentation enables precise boundary delineation, volumetric quantification, and therapy response assessment. These tasks directly inform clinical management strategies, including surgical resection margins, radiation targeting, and chemotherapy regimen optimization [1,2], while manual segmentation performed by expert radiologists provides high-quality results, it is labor-intensive, time-consuming, and highly dependent on the expertise of the annotator. As the volume of medical imaging data increases due to advances in imaging technologies and the need for more frequent monitoring, manual segmentation becomes increasingly impractical. Furthermore, in regions with limited access to specialized medical personnel, manual segmentation is often not feasible, exacerbating the problem in clinical practice.

In response to these challenges, automatic brain tumor segmentation using deep learning (DL) techniques has become an active area of research. Deep learning-based methods, particularly convolutional neural networks (CNNs), have shown remarkable success in medical image segmentation tasks, outperforming traditional image processing techniques [3,4,5,6,7,8,9,10]. These methods are capable of automatically learning hierarchical feature representations from raw image data, making them more adaptable to diverse segmentation tasks involving different anatomical structures and imaging modalities. In particular, U-Net [11] and its variants [12,13,14,15] have become the go-to models for medical image segmentation due to their ability to work effectively with relatively small datasets, a common limitation in medical imaging.

Despite the successes of CNN-based models, several challenges remain in the field of medical image segmentation. One of the most significant hurdles is the lack of large, diverse annotated datasets. Medical image segmentation models are typically trained on limited datasets, which can lead to over-fitting and poor generalization [16,17]. Additionally, medical images often suffer from variations in acquisition conditions (e.g., scanner type, acquisition protocol) that can make it difficult for models trained on one dataset to generalize to another. To mitigate these issues, researchers have turned to transfer learning and pre-trained models [18] to leverage larger, more diverse datasets, such as those from natural images, as a starting point.

The recent introduction of the “Segment Anything Model” (SAM) has demonstrated significant potential in general image segmentation tasks, though its clinical applicability requires further validation. SAM is a foundation model trained on an enormous dataset of 11 million images and 1 billion masks, which provides it with significant versatility. SAM can handle multiple types of input prompts, including points, boxes, and text, making it highly adaptable to a wide range of segmentation tasks [19]. However, despite its success in natural image segmentation, SAM has limitations when applied to medical images, particularly due to the substantial differences between natural and medical image modalities. Medical images, such as MRI scans, contain complex structures with significant variations in anatomical and pathological features across patients, which SAM may not be equipped to handle directly [20].

To address these challenges, a medical variant of SAM, called MedSAM, was developed [21]. MedSAM is fine-tuned on a large-scale medical dataset consisting of over a million annotated medical images, and it shows significant improvements over the original SAM model when applied to medical image segmentation tasks. By leveraging domain-specific features and anatomical priors, MedSAM performs better in tasks such as brain tumor segmentation, where multi-modal data is often required to capture all relevant features. Despite these advances, the application of SAM-based models to multi-modal medical imaging remains an open challenge, particularly when dealing with missing modalities.

To effectively apply the SAM model to multimodal data, effective feature fusion strategies play a crucial role in improving the performance of multi-modal segmentation models. Feature fusion methods aim to combine complementary information from multiple modalities in a way that enhances the model’s ability to make accurate predictions. Attention mechanisms have proven to be particularly effective in this regard. For instance, the Squeeze-and-Excitation (SE) module [22] allows the network to adaptively recalibrate feature channels, while cross-attention mechanisms can focus on the most relevant features for segmentation. These methods help ensure that the network learns to prioritize the most informative features from each modality, which is particularly beneficial when working with multi-modal MRI data [23].

In clinical practice, MRI data is often incomplete due to missing modalities (e.g., T1, T2, FLAIR, or T1gd), which may result from image corruption, artifacts, acquisition protocols, patient allergies to contrast agents, or cost considerations [24]. This poses a significant challenge for multi-modal learning, where the model needs to handle missing data during training and inference without compromising the segmentation performance. Several strategies have been proposed to address the missing modality problem. One approach is to simply replace missing modalities with zeros or random values [25], though this does not provide meaningful information and may result in poor performance. More advanced methods aim to synthesize the missing modality using generative models [26,27], but generating high-quality synthetic data remains a significant challenge. Another approach is to rely on feature sharing between modalities, where models learn shared representations from existing modalities to minimize the loss of information due to missing data [23].

This paper proposes a multi-modal SAM model (MSAM) for brain tumor segmentation, with the following key contributions:

1. Extension of SAM to Multi-Modal MRI Data: We extend the SAM model to work with multi-modal MRI data, experimentally validating the effectiveness of different modality combinations for tumor segmentation. Our results show that structural MRI modalities (T1, T2, T1gd, FLAIR) significantly improve segmentation performance, particularly for enhancing tumors segmentation.
2. Robust Feature Fusion Strategy: We propose a robust feature fusion strategy within the MSAM framework, which is capable of integrating information from multiple modalities effectively. The model is able to maintain high segmentation accuracy even when one modality is missing, making it more adaptable to real-world clinical scenarios where modality availability is often inconsistent.
3. Missing Modality Training Strategy: We introduce a novel training strategy that improves the model’s ability to handle missing modalities. By simulating missing modalities during training, our model learns to compensate for the absence of critical information and achieves improved performance in real-world scenarios where data may be incomplete.

By addressing the challenges posed by multi-modal data and missing modalities, MSAM advances the state-of-the-art in brain tumor segmentation and demonstrates the potential of SAM-based models for medical image analysis.

## 2. Methods

### 2.1. Introduction to SAM

The Segment Anything Model (SAM), proposed by Meta AI, is an advanced image segmentation model consisting of three core components: the image encoder, the prompt encoder, and the mask decoder. The image encoder transforms the input image into feature maps, the prompt encoder encodes various types of prompts (such as points, boxes, text, etc.), and the decoder generates the segmentation mask for the input image based on the image feature embeddings and prompt encoding embeddings.

**Figure 1:**
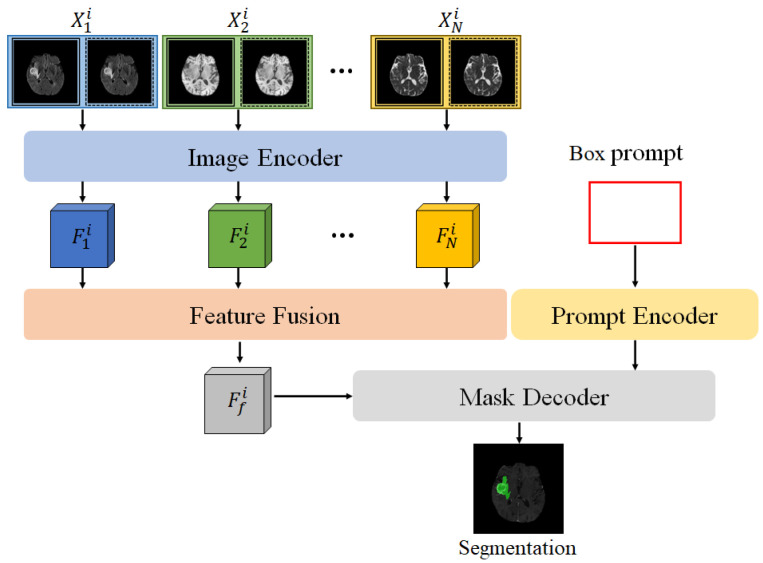
Structure of the multi-modal SAM model (MSAM).

In the multi-modal SAM model (MSAM) proposed in this paper, the SAM framework is extended, as shown in Figure 1. Unlike the original SAM, MSAM first applies the encoder to perform feature encoding on multi-modal MRI data ($X_{1},X_{2},\ldots ,X_{N}$) to obtain feature representations for each modality, denoted as $F_{1},F_{2},\ldots ,F_{N}$. To effectively fuse the multi-modal features and adapt them for SAM’s mask decoder, we design a Feature Fusion Block (FFB), which combines the multiple feature maps $F_{1},F_{2},\ldots ,F_{N}$ into a comprehensive multi-modal fused feature $F_{f}$.

### 2.2. Feature Fusion Block

The Feature Fusion Block aims to integrate multi-modal features from the SAM encoder, emphasizing tumor-relevant features and enhancing segmentation accuracy in the decoder. Common fusion techniques, such as average and max pooling, are limited: average pooling may dilute important features, while max pooling overlooks subtle yet crucial information.

**Figure 2:**
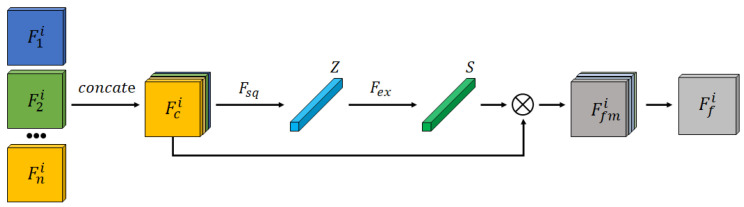
The structure of the Feature Fusion Block (FFB).

The Feature Fusion Block structure is depicted in Figure 2. Encoded features from different modalities are first concatenated and input into a channel attention mechanism to generate a fused feature representation, $F_{fm}^{i}$. This recalibrates feature weights to enhance tumor segmentation accuracy.

To effectively capture the inter-dependencies between different feature channels and optimize fusion outcomes, a channel attention mechanism is incorporated into the Feature Fusion Module, as illustrated in Figure 2. Specifically, the different modality features extracted by the encoder, $\left[F_{1}^{i},F_{2}^{i},\ldots ,F_{N}^{i}\right]$, where $F_{n}^{i}\in R^{C_{1}\times H\times W}$, are concatenated into a new feature map $F_{c}^{i}\in R^{C_{2}\times H\times W}$. Global Average Pooling is then applied over the spatial dimensions H×W of the feature map to generate a weight vector $Z\in R^{C_{2}\times 1\times 1}$, where the *k*-th element is calculated as follows:

(1) $$z_{k}=F_{sq}\left(F_{c}^{i}\left(k\right)\right)=\frac{1}{H\times W}\sum_{h=1}^{H}\sum_{w=1}^{W}F_{c}^{i}\left(k,h,w\right)$$

Next, a two-layer fully connected network captures channel correlation:

(2) $$s=F_{ex}\left(z,W\right)=\sigma \left(g(z,W)\right)=\sigma \left(W_{2}\delta \left(W_{1}z\right)\right)$$

where δ is the ReLU activation function, σ denotes Sigmoid, and $W_{1},W_{2}$ are weight matrices. This network generates channel attention weights *s*, applied as follows:

(3) $$F_{fm}^{i}=s\cdot F_{c}^{i}$$

The final fused feature map $F_{f}^{i}$ is then obtained using a residual layer, yielding a comprehensive feature representation optimized for segmentation.

This fusion approach effectively combines features from different modalities, generating a more expressive fused feature representation than traditional mean or max fusion methods, thereby significantly improving segmentation performance.

### 2.3. Training and Prediction Methods

In multi-modal model training, the conventional approach is referred to as Full Modality Training (FT). To better handle segmentation tasks with missing modalities, we propose a specialized training method called Missing Modality Training (MT). The Full Modality Training (FT) approach trains on all modalities, whereas the Missing Modality Training (MT) approach adapts the model to perform under conditions of missing data, enhancing its robustness.This approach significantly improves tumor segmentation performance under missing modality conditions without altering the model structure. Through MT training, the Feature Fusion Block can more effectively extract features from available modalities, thereby providing more stable and reliable segmentation features to the subsequent decoder.

For data with missing modality $X\in R^{C\times H\times W}$, we construct substitute data $\bar{X}\in R^{C\times H\times W}$, where each element ${\bar{x}}_{c,h,w}$ is independently sampled from a uniform distribution between 0 and 1, satisfying:

(4) $$\begin{array}{cc} {\bar{x}}_{c,h,w} & ∼U(0,1)\\ \forall \;c & \in \{1,2,\ldots ,C\},\;\\ h & \in \{1,2,\ldots ,H\},\;\\ w & \in \{1,2,\ldots ,W\} \end{array}$$

This random substitution aids the model in generalizing across different missing modality scenarios by providing surrogate data for absent features.

During training, when a modality $X_{n}$ is missing, it is not completely replaced by the randomly generated ${\bar{X}}_{n}$; instead, it is replaced based on a probability λ∈[0,1], as follows:

(5) $$X_{in}=\left\{\begin{array}{cc} X_{n} & p\leq \lambda \\ {\bar{X}}_{n} & p>\lambda \end{array}\right.$$

Here, λ determines the likelihood of using real versus randomly generated data for a missing modality, enabling controlled adaptation to partial modality information.

In model prediction, we differentiate between Full Modality Prediction (FP) and Missing Modality Prediction (MP). Under FI mode, the model receives input from all modalities; in MI mode, any missing modality $X_{n}$ is entirely replaced by the corresponding random data ${\bar{X}}_{n}$ to ensure the model’s adaptability to missing modalities.

### 2.4. Compared Methods

For brain tumor segmentation, several state-of-the-art deep learning approaches have been proposed, including nnU-Net, UNETR, and SwinUNETR, which have demonstrated promising results in medical image segmentation tasks.

nnU-Net (no-new-U-Net) is a fully automated and task-independent framework designed to achieve high performance in medical image segmentation [14]. nnU-Net adapts its architecture, preprocessing pipeline, and training strategy based on the characteristics of the dataset, including the image modality, size, and the number of classes. This adaptability has allowed nnU-Net to outperform many other methods in various challenges without requiring task-specific fine-tuning. Its robustness and simplicity make it a reliable choice for a wide range of segmentation problems, including brain tumor segmentation in multi-modal MRI datasets.

UNETR (U-Net Transformer) leverages the strengths of both U-Net and Transformer architectures [28]. By replacing the conventional convolutional blocks in U-Net with self-attention mechanisms from transformers, UNETR is capable of capturing long-range dependencies in the image, making it particularly effective for volumetric data such as MRI scans. The model excels in segmenting complex structures by learning global context and fine-grained local details simultaneously. This hybrid architecture has proven to be effective in various medical imaging tasks, including brain tumor segmentation.

SwinUNETR (Swin Transformer U-Net) integrates the Swin Transformer, a hierarchical vision Transformer that uses shifted windows for efficient image processing, with the U-Net architecture [29]. This approach takes advantage of the Swin Transformer’s ability to learn both global and local contextual information through hierarchical representations, enhancing segmentation performance in complex medical imaging tasks. SwinUNETR has demonstrated superior performance in brain tumor segmentation, especially when using multi-modal MRI data, by capturing both fine details and high-level features in the images.

These methods represent the cutting edge of deep learning architectures in medical image segmentation, each combining different strengths of convolutional networks, transformers, and self-attention mechanisms to improve the accuracy and efficiency of brain tumor segmentation.

## 3. Experiment

### 3.1. Dataset and Preprocessing

In this study, we utilize the UPenn-GBM dataset, a publicly available, preprocessed multiparametric MRI (mpMRI) dataset curated by the University of Pennsylvania Health System and hosted by The Cancer Imaging Archive [30,31,32]. The dataset comprises 3D MRI scans from 611 individual subjects, with different modalities: (a) structural modalities: T1 (native T1-weighted), T1gd (contrast-enhanced T1), T2 (native T2-weighted), and FLAIR (Fluid-Attenuated Inversion Recovery); (b) Diffusion Tensor Imaging (DTI) modalities: tensor tracks (DTI-TR), axial diffusivity (DTI-AD), radial diffusivity (DTI-RD), and fractional anisotropy (DTI-FA). Preprocessing steps included skull stripping and image co-registration to align the MRI modalities.

A subset of this dataset, the BraTS challenge dataset [33], contains 173 samples and serves as a benchmark for glioma segmentation, making it particularly suitable for evaluating the proposed method. Additionally, the UPenn-GBM dataset provides segmentation masks for tumor regions, including Necrotic Core (NCR), Edema (ED), Enhancing Tumor (ET), and healthy tissue (Else). Among the 611 subjects, 147 have expert-annotated segmentation masks, which were used as ground truth for model training.

To prepare the data for model input, we applied the following preprocessing pipeline:

1. Gray-Level Adjustment: Based on the histogram of gray values in the original images, we removed voxels with gray values below the 0.5% and above the 99.5% percentiles, subsequently linearly scaling the retained voxel intensities to a 0–255 range.
2. 3D to 2D Conversion: Following the ground truth, we selected tumor-containing slices along the Z-axis, converting the data into a series of 2D images.
3. Data Normalization: To meet the model input requirements, all images were resized to 1024×1024 pixels. Since the SAM model requires a three-channel RGB image format, we replicated the grayscale data across three channels, normalizing the pixel intensity values to a range of 0–1.

These preprocessing steps ensure that the 3D MRI data were transformed into a suitable format for the multi-modal SAM model, preserving both structural and functional information relevant for glioma segmentation.

### 3.2. Implementation Details

Our method is implemented using the PyTorch 2.6 framework. The encoder, decoder, and prompt encoder of the SAM model are initialized with the pre-trained weights from MedSAM [21]. During training, the parameters of the image encoder and prompt encoder remain frozen, while the parameters of the mask decoder and feature fusion module are updated. Bounding box prompts are extracted from expert-annotated masks and perturbed randomly within a 0–20 pixel range to enhance model robustness.

The model is trained using the AdamW optimizer, with an initial learning rate set to 0.0001 and a weight decay coefficient of 0.01. The batch size is set to 8, and training is conducted for 50 epochs. All training sessions are performed on an Nvidia V100 GPU with 32 GB of memory. To ensure model robustness and generalization, we use 5-fold cross-validation across the entire dataset. For the missing modality training, the value of λ is set to 0.5.

### 3.3. Loss Function

In our approach, the final loss function is defined as the sum of the Dice loss and binary cross-entropy (BCE) loss to optimize the model’s segmentation performance.

Dice Loss: Measures the overlap between the predicted segmentation and the ground truth, encouraging the model to maximize the similarity between the two. It is defined as:

(6) $$L_{\mathrm{Dice}}=1-\frac{2\sum_{i=1}^{N}y_{i}\hat{y_{i}}}{\sum_{i=1}^{N}y_{i}^{2}+\sum_{i=1}^{N}{\hat{y_{i}}}^{2}}$$

where *N* denotes the total number of voxels, and $y_{i}$ and $\hat{y_{i}}$ represent the ground truth and predicted values for voxel *i*, respectively.

Binary Cross-Entropy (BCE) Loss: Measure the pixel-wise classification error, defined as:

(7) $$L_{\mathrm{BCE}}=-\frac{1}{N}\sum_{i=1}^{N}\left[y_{i}\log\hat{y_{i}}+\left(1-y_{i}\right)\log\left(1-\hat{y_{i}}\right)\right]$$

The total loss function, $L_{\mathrm{total}}$, is then defined as the sum of the Dice loss and binary cross-entropy loss:

(8) $$L_{\mathrm{total}}=L_{\mathrm{Dice}}+L_{\mathrm{BCE}}$$

### 3.4. Evaluation Metrics

To quantitatively evaluate the proposed method, we employ two widely used segmentation metrics: the Dice Similarity Coefficient (Dice Score) and the 95% Hausdorff Distance (HD95).

Dice Similarity Coefficient: The Dice Score measures the degree of overlap between the predicted segmentation and the ground truth, ranging from 0 to 1, where a value closer to 1 indicates better prediction accuracy. The Dice Similarity Coefficient is defined as:

(9) $$\mathrm{Dice}\;\mathrm{Score}=\frac{2\left|G\cap S\right|}{\left|G\right|+\left|S\right|}$$

where *G* denotes the ground truth segmentation region, and *S* represents the predicted segmentation region.

95% Hausdorff Distance (HD95): The HD95 metric assesses the similarity between two point sets and is a common distance metric in image segmentation tasks. It describes the largest distance between two point sets by computing the distance from each point in one set to the nearest point in the other set and taking the maximum of these values in both directions. Specifically, HD95 refers to the 95th percentile of the Hausdorff Distance, which helps mitigate the impact of outliers. The HD95 is defined as:

(10) $$\begin{array}{c} H\left(G,S\right)=\max\left(\max_{g\in G}\left\{\min_{s\in S}\left\|s-g\right\|\right\},\right.\\ \left.\max_{s\in S}\left\{\min_{g\in G}\left\|g-s\right\|\right\}\right) \end{array}$$

where *G* and *S* denote the sets of points in the ground truth and predicted segmentation results, respectively, with $G=\{g_{1},g_{2},\ldots ,g_{N}\}$ and $S=\{s_{1},s_{2},\ldots ,s_{N}\}$.

Both metrics provide comprehensive insights into the model’s ability to capture tumor regions, with the Dice Score reflecting overall overlap and the HD95 capturing boundary precision.

## 4. Results

To assess the performance of the proposed MSAM model, we conducted a series of comparative experiments, focusing on multi-modal glioma segmentation tasks. These experiments aimed to evaluate the effectiveness of different modality combinations, as well as compare the segmentation performance of MSAM with that of the widely-used U-Net, nnU-Net, UNETR, and SwinUNETR.

### 4.1. Segmentation Performance

We first investigated the impact of different modality combinations on segmentation performance, particularly the integration of structural modalities and Diffusion Tensor Imaging (DTI) modalities. The goal was to identify the optimal combination of modalities for glioma segmentation.

**Figure 3:**
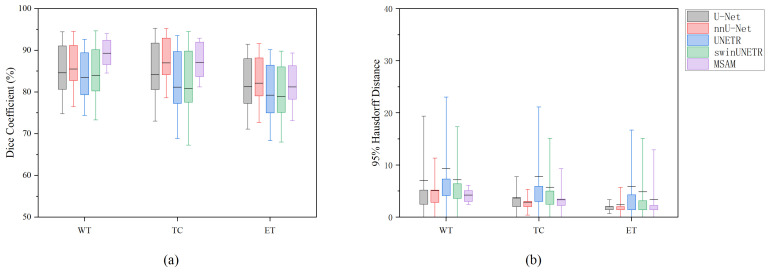
Segmentation results across different methods for various tasks under structural modalities. (**a**) Dice score results, (**b**) HD95 distance results. Each box represents the mean and standard deviation for each segmentation task across six different segmentation methods.

Subsequently, we compared the performance of MSAM with U-Net under various modality combinations. The segmentation performance was evaluated using two key metrics: Dice Similarity Coefficient (DSC) and 95% Hausdorff Distance (HD95). The suffix “-SD” indicates that the method uses both structural modalities and DTI modalities; the suffix “-S” and “-D”indicates that the method just uses the structural Modalities and DTI modalities, respectively. The results are summarized in Table 1 and Figure 3.

Under identical modality input conditions, MSAM consistently outperformed U-Net, UNETR, and SwinUNETR add across all three segmentation tasks, as measured by both DSC and HD95. However, for TC and ET segmentation, MSAM performs slightly worse than nnU-Net. Specifically, for the Whole Tumor (WT) and Tumor Core (TC) regions, MSAM exhibited superior segmentation accuracy. The performance difference in the Enhancing Tumor (ET) region was relatively smaller, but MSAM still showed an edge, particularly under the DTI modality condition (MSAM-D). While both models performed poorly when only DTI data were used, MSAM showed a clear advantage in segmenting the ET region under this condition.

The standard deviation of MSAM’s segmentation results was notably lower than that of the compared methods, indicating greater stability and reliability in tumor segmentation tasks. These findings suggest that MSAM provides more consistent and robust performance compared to the others, especially for the segmentation of ET regions.

The experimental results further revealed that the choice of modality input significantly influences segmentation performance. For the WT and TC segmentation tasks, MSAM-S achieved the highest DSC of 89.27% and 87.05%, respectively, with HD95 values of 4.24 and 3.34. For the ET segmentation task, MSAM-S achieved the highest DSC of 81.23%, while MSAM-SD and MSAM-D showed a comparable HD95 value of 2.00. Overall, MSAM-S demonstrated the best performance across all tasks.

When only DTI modality data were used, segmentation performance was poor, particularly for the TC and ET tasks. The performance of the methods under the DTI-only condition was suboptimal, with both models showing significant degradation in segmentation accuracy. This highlights the importance of integrating structural modalities for accurate tumor segmentation.

**Figure 4:**
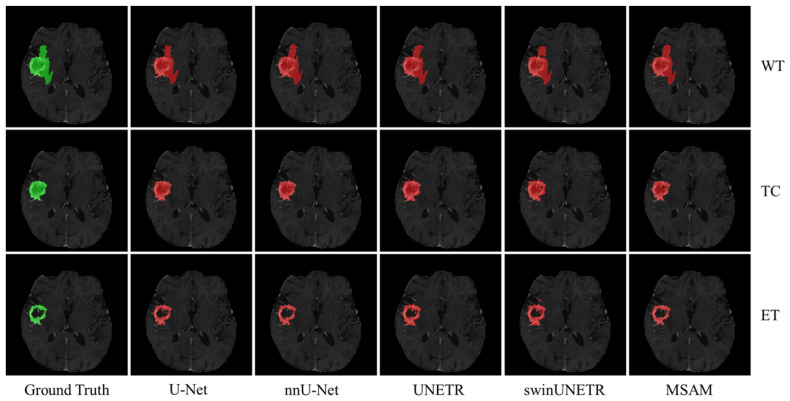
Segmentation results for the three segmentation tasks when only structural modality data are used as input. In the figure, green denotes ground truth while red indicates the corresponding segmentation mask.

Figure 4 presents qualitative results for the three segmentation tasks using structural modality data as input. The green regions represent the ground truth, while the red areas indicate the corresponding segmentation masks. As shown, MSAM-S’s segmentation results are much closer to the ground truth, particularly in capturing finer details, compared to U-Net-S and UNETR-S, that demonstrate less precise delineation of tumor boundaries. This finding is consistent with the quantitative results summarized in Table 1.

### 4.2. Evaluation of FFB

We concurrently evaluated the utility of the FFB framework by performing tumor sub-region segmentation using single-modality data. As shown in Table 2, the comparative results of different fusion strategies are presented in terms of Dice Similarity Coefficient (DSC) and 95th percentile Hausdorff Distance (HD95).

Using the multimodal fusion model MSAM-S as the baseline, systematic comparisons with eight single-modality models (e.g., SAM-FLAIR using only FLAIR sequences, SAM-T1gd using only T1gd sequences) and two degraded fusion variants (MSAM-SD/MSAM-D) demonstrated the significant advantages of multimodal fusion. The experiments revealed comprehensive performance improvements across all tasks for the multimodal model: Compared to single-modality approaches, MSAM-S achieved a maximum DSC increase of 1.1% (81.23% vs. SAM-T1gd’s 80.13%) in ET segmentation while maintaining a mean HD95 of 2.00 mm, representing a 51.7% improvement in boundary precision over the best single-modality method (SAM-T1gd: 4.14 mm).

Notably, substantial performance variations were observed among single-modality models across different tasks. For instance, while SAM-T1gd demonstrated optimal ET segmentation (DSC = 80.13%), its TC segmentation performance significantly deteriorated (DSC = 85.70% vs. the multimodal baseline of 87.05%). Similarly, SAM-FLAIR achieved 88.88% DSC in WT segmentation but failed to effectively delineate ET regions (DSC = 58.57%). These task-specific sensitivity disparities underscore the critical importance of multimodal complementarity: through feature fusion mechanisms, MSAM-S successfully integrates anatomical information from different imaging sequences, achieving optimal performance balance across all three segmentation tasks.

Further analysis revealed that the degraded model MSAM-D (with spatial alignment module removed) exhibited catastrophic performance degradation in TC segmentation, with DSC plummeting by 17.1 percentage points to 69.95% and HD95 error expanding to 22.05 mm (a 558% increase from baseline). The single-modality model SAM-ad completely failed in complex region segmentation (ET: DSC = 48.85%, HD95 = 36.36 mm), providing counterfactual validation of multimodal necessity. The experiments also exposed inherent limitations of single-modality methods: SAM-T2 produced 11.20 mm HD95 errors in TC segmentation (3.35× the baseline), while SAM-rd showed outlier HD95 values of 25.82 mm in ET segmentation. In contrast, the multimodal approach reduced these errors to clinically acceptable ranges through cross-modal feature calibration.

### 4.3. Evaluation of Missing Modality Training

We also explored the effect of missing modalities on segmentation performance by training MSAM under conditions where specific modalities were absent. To further enhance model performance under missing modality conditions, a Missing Modality Training (MT) mode was adopted. Table 3 reports the Dice Score results for various prediction modes under different missing modality conditions. The modes include Full Modality Training and Full Modality Prediction (FTFP), Missing Modality Training and Full Modality Prediction (MTFP), Full Modality Training and Missing Modality Prediction (FTMP) and Missing Modality Training and Missing Modality Prediction (MTMP).

From Table 3, it is evident that within the same training mode, whether Full Modality Training (FT) or Missing Modality Training (MT), Full Modality Prediction (FI) generally surpasses Missing Modality Prediction (MP) in segmentation performance. Under the FT mode, the FP segmentation results consistently exceed those of MP; in the MT mode, the three FP results (MTFP-T1 for TC, MTFP-FLAIR and MTFP-T1 for ET) are slightly lower than those of MP, but the differences are minimal and not statistically significant after significance testing. These findings indicate that full modality input significantly enhances brain tumor segmentation performance, with each modality playing a crucial role in the segmentation task.

Under Full Modality Prediction (FP), FTFP surpasses MTFP only in the WT segmentation task, with a Dice Score of 89.27%, while MTFP-T2 and MTFP-FLAIR achieve the highest Dice Scores in the TC and ET segmentation tasks, at 87.36% and 81.28%, respectively.

Under Missing Modality Prediction (MP), MTMP outperforms FTMP in the vast majority of cases (11/12), with the sole exception being the WT segmentation task where MTMP-T1’s result is slightly lower than that of FTFI-T1. Different modalities hold varying levels of importance for different tumor regions, with the FLAIR modality being particularly critical for WT segmentation, while the T1gd modality is essential for TC and ET segmentation. The absence of these key modalities significantly degrades the corresponding segmentation results. However, the findings reveal that the Missing Modality Training (MT) mode effectively mitigates the adverse effects of missing key modalities, substantially enhancing Dice Scores by 6.05%, 6.79%, and 18.02%, respectively.

We further validate the effectiveness of the proposed modality fusion module by performing feature visualizations, allowing an intuitive analysis of the differences in feature representation across various training modes and input modalities. To achieve this, we employed Uniform Manifold Approximation and Projection (UMAP), a nonlinear dimensionality reduction technique that maps high-dimensional data into a lower-dimensional space while preserving both local and global structures. UMAP is particularly effective in data visualization and feature extraction, offering high efficiency for large datasets and strong preservation of global data relationships.

**Figure 5:**
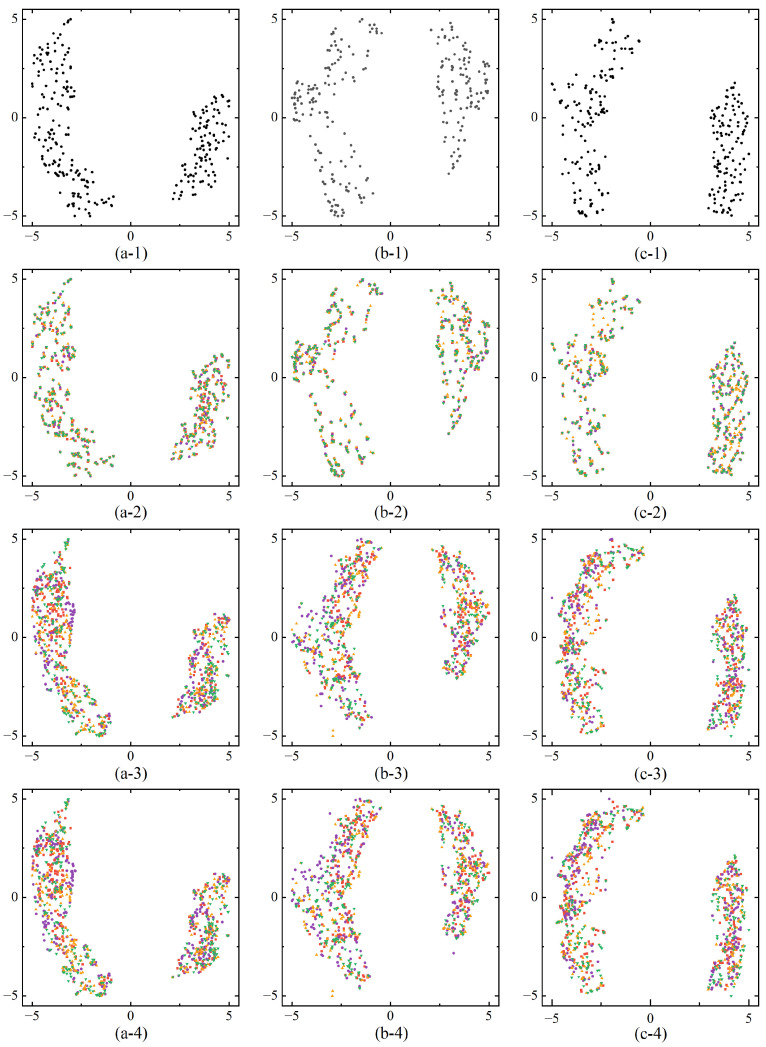
Feature visualizations after feature fusion across different training modes and input modalities. (**a**) Whole Tumor segmentation task features, (**b**) Tumor Core segmentation task features, (**c**) Enhancing Tumor segmentation task features. The labels 1-FTFI, 2-FTMI, 3-MTFI, and 4-MTMI represent different modality input configurations. Red squares indicate missing FLAIR, purple dots indicate missing T1, orange triangles indicate missing T1gd, and green inverted triangles indicate missing T2.

Figure 5 presents feature visualizations after modality fusion under different training modes and input modalities. In the three segmentation tasks (Whole Tumor, Tumor Core, Enhancing Tumor), the features distinctly separate into foreground and background domains, visually demonstrating that the MSAM model can effectively distinguish between tumor and non-tumor regions. Under the same training mode, the feature distributions after fusion exhibit a similar pattern regardless of whether full or missing modality input is used. However, in the Full Modality Training (FT) mode, when certain modalities are absent during prediction, the feature distributions across modalities overlap substantially. This overlap occurs because the model optimizes for full-modality input during training, meaning that the absence of specific modalities during prediction does not significantly affect the feature distributions of the remaining modalities. This suggests that if the missing modality is not crucial to the segmentation task, the impact on performance is minimal. However, if a key modality is absent, segmentation accuracy is notably reduced. Therefore, the FT training mode is somewhat rigid in addressing missing modalities, as it lacks the flexibility to compensate for missing information and struggles to leverage the available modalities to accurately segment tumor regions.

In contrast, the feature distributions under the Missing Modality Training (MT) mode exhibit a lower degree of overlap and are more distinct compared to the FT mode. This indicates that the MT mode can adapt more effectively to missing modality conditions by making specific adjustments, thereby compensating for the absence of certain modalities. As a result, segmentation performance improves significantly when key modalities are missing, as demonstrated by the results in Table 3.

### 4.4. Impact of Hyperparameter in Missing Modality Training

**Figure 6:**
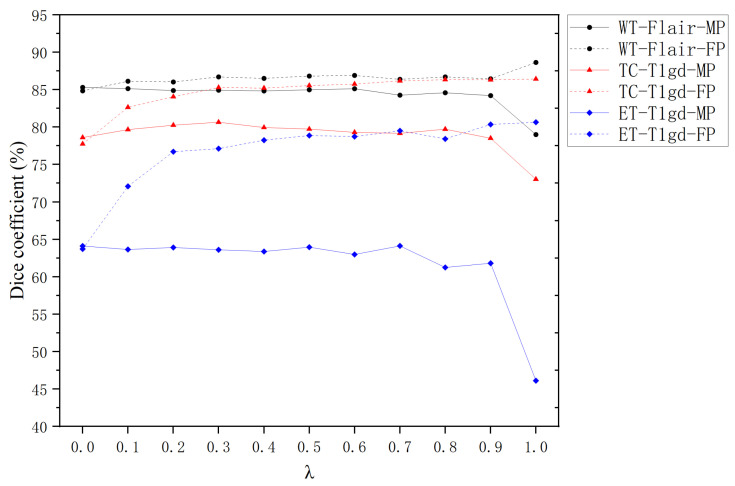
Dice Coefficient variations with respect to λ values. Solid/dashed lines represent MP/FP modes, respectively. •: WT-FLAIR, ▴: TC-T1gd, and ⧫: ET-T1gd.

The hyperparameter λ (ranging from 0 to 1) plays a pivotal role in governing the behavior of Missing Modality Training (MT). Building on the findings from Section 4.3 and prior studies [33], which highlight the modality-specific importance of FLAIR sequences for Whole Tumor segmentation and T1gd for Tumor Core and Enhancing Tumor delineation, we systematically evaluate the influence of λ under targeted modality absence conditions. Figure 6 illustrates the Dice coefficients achieved by Full Modality Prediction (FP) and Missing Modality Prediction (MP) strategies across different λ values for individual segmentation tasks.

For all segmentation tasks (WT, TC, ET), FP consistently demonstrates superior performance to MP when λ is within the range of (0,1). This aligns with the expectation that FP benefits from richer feature representations, enabling more precise segmentation. The magnitude of improvement follows the hierarchy:

(11) $$\Delta {\mathrm{Dice}}_{\mathrm{ET}-T1\mathrm{gd}-\mathrm{FP}}>\Delta {\mathrm{Dice}}_{\mathrm{TC}-T1\mathrm{gd}-\mathrm{FP}}>\Delta {\mathrm{Dice}}_{\mathrm{WT}-\mathrm{FLAIR}-\mathrm{FP}}$$

where ΔDice represents the performance gain relative to MP baseline.

At λ=0, MP shows marginal superiority over FP. This counterintuitive result stems from complete modality replacement with random noise during training, creating inference interference when actual modalities are reintroduced. When λ=1, the system converges to Full Training (FT) mode, where the mean Dice Score of FP significantly outperforms MP’s.

Within the practical range of 0<λ<1, MP mode shows limited sensitivity to λ variations. For WT-FLAIR-MP and ET-T1gd-MP, a gradual decline in Dice Scores is observed as λ increases, suggesting that higher λ values may amplify interference from incomplete modality information. In contrast, TC-T1gd-MP displays a non-monotonic trend, with peak performance occurring at λ=0.7. For FP mode, all tasks exhibit a consistent upward trend in Dice Scores with increasing λ, underscoring the robustness of MT in mitigating modality absence during inference.

The experimental results validate that MT effectively enhances model robustness against missing modalities during inference. The observed patterns support the selection of λ=0.5 as an optimal compromise, balancing feature preservation and noise suppression across all tasks. This configuration achieves a mean Dice Coefficient of 0.84 ± 0.03, demonstrating balanced performance without significant task-specific bias. The findings emphasize the importance of task-aware parameter tuning in multimodal segmentation frameworks.

### 4.5. Statistical Analysis of Tumor Regions

**Figure 7:**
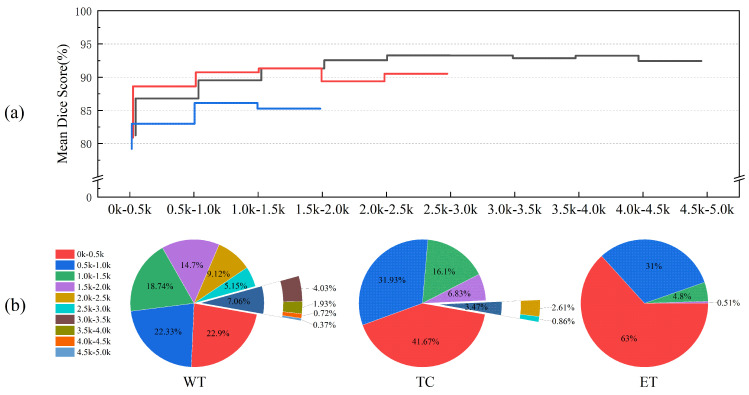
Statistics of tumor regions of different sizes (2D). (**a**) Dice Scores for segmenting tumor regions of different sizes, with the x-axis representing the number of pixels in the tumor region. The black lines represent the Whole Tumor (WT), red lines represent the Tumor Core (TC), and blue lines represent the Enhancing Tumor (ET). (**b**) Proportion of different-sized tumor regions relative to the total sample count.

We also analyzed the impact of tumor region size on segmentation performance and present the results for tumor regions of various sizes in Figure 7. As the tumor region size increases, the Dice Score initially increases, reaching a peak before slightly declining. This trend suggests that segmentation performance is strongly influenced by the size of the tumor regions, with smaller regions resulting in lower segmentation accuracy. Specifically, for the Whole Tumor (WT), Tumor Core (TC), and Enhancing Tumor (ET) tasks, the Dice Scores for tumor regions in the 0–0.5 k voxel size range are 81.24%, 80.89%, and 79.21%, respectively, representing the lowest values.

The size distribution of the target regions varies across the segmentation tasks, as shown in Figure 7b. The WT size range spans 0–5.0 k voxels, with TC, being a sub-region of WT, ranging from 0 to 3.0 k voxels, and the ET region falling within the 0–2.0 k voxel range. In the 0–2.0 k size range, segmentation performance for the ET region is generally lower than that for the WT and TC regions. Furthermore, TC performance in the 0–0.5 k size range is slightly inferior to that of WT but surpasses WT in other intervals. Given the target region size distribution for each task, we conclude that the ET region, being smaller and more challenging to segment, leads to poorer segmentation performance compared to the WT and TC regions.

## 5. Discussion

In this study, we comprehensively evaluated the MSAM model for multimodal brain tumor segmentation under various challenging conditions, including missing imaging modalities and varying tumor region sizes. The results consistently demonstrate MSAM’s robustness, its ability to adapt to incomplete input data, and its superior performance across multiple segmentation tasks. In this section, we interpret the major findings, contrast them with related work, and discuss their implications.

Our findings underscore the critical role of multimodal fusion in achieving accurate and robust brain tumor segmentation. MSAM-S, which integrates structural MRI modalities, achieved the good performance, particularly in segmenting the Enhancing Tumor (ET), a region characterized by irregular morphology and unclear boundaries. Structural modalities such as T1, T1gd, T2, and FLAIR were particularly effective for segmenting larger tumor regions like Whole Tumor (WT) and Tumor Core (TC), as evidenced by the strong performance of MSAM-S. These results highlight that different modalities contribute complementary information for accurate tumor region segmentation. The fusion of these heterogeneous features enhances the SAM’s ability to delineate both large and small tumor regions. This indicates that combining different imaging sequences is very important for improving the segmentation performance of complex tumor subregions. Future research will explore advanced fusion methodologies—notably hybrid attention architectures and hierarchical multi-scale integration—to model complex inter-modal dependencies. Such approaches may enhance information synthesis across extended modality sets, potentially elevating performance in clinically heterogeneous brain tumor segmentation tasks.

Segmentation results under different modalities missing further emphasize the importance of key modalities. When all modalities were available, MSAM achieved optimal results, particularly for WT and TC. However, the absence of critical modalities—especially T1gd and T2—led to significant performance degradation in ET segmentation, suggesting that these modalities are essential for capturing the complex contrast and boundary information associated with the ET region. This is consistent with prior reports on the relevance of contrast-enhanced and fluid-sensitive sequences in tumor delineation [23,33].

A key contribution of this study is the introduction of a Missing Modality Training (MT) strategy. Instead of attempting to reconstruct missing inputs, MT allows the model to learn robust representations using only the available modalities during training. This is achieved by substituting missing modalities with noise drawn from a uniform distribution to serve as placeholders, preserving the structural integrity of the network. Our empirical observations confirm that MSAM trained with MT can dynamically adjust feature distributions in the latent space according to modality availability, thus achieving stable and reliable segmentation performance even under missing data conditions, while more complex methods such as generative reconstruction could potentially provide more accurate modality estimation [25], our goal was to evaluate the MSAM architecture’s intrinsic ability to generalize under incomplete inputs. Future work may explore the integration of generative or modality-aware imputation techniques as complementary modules.

Despite the overall effectiveness of our approach, we observed a marked drop in segmentation accuracy for small tumor regions, particularly in the ET task. Quantitative analysis revealed that tumors with fewer voxels (e.g., <0.5 k) exhibited significantly lower Dice Scores. This phenomenon likely stems from limited spatial context, low inter-region contrast, ambiguous boundaries, and an imbalanced representation of small lesions in the training set. Such limitations are common in medical image segmentation and highlight the need for targeted solutions.

To address this challenge, future research could incorporate region-aware loss functions that emphasize underrepresented tumor sizes, utilize resolution-preserving or multi-scale architectures to capture fine-grained features, and apply attention-based mechanisms that can selectively focus on ambiguous or subtle tumor regions. These enhancements may improve segmentation performance for small, complex lesions and further increase the clinical utility of the MSAM model.

## 6. Conclusions

In this study, we introduced a multi-modal segmentation framework that integrates different modalities for accurate glioma tumor segmentation. The experimental results indicate that MSAM significantly outperforms U-Net in segmentation accuracy and stability, whether under full-modality or missing-modality conditions. The feature visualization analysis reveals that MSAM is adept at distinguishing tumor from non-tumor regions, even when some modalities are missing, highlighting its robustness and flexibility. Additionally, we demonstrated that tumor size impacts segmentation performance, with smaller regions posing more challenges. These results suggest that MSAM offers a promising solution for improving glioma segmentation in clinical settings, especially when dealing with incomplete data or varying tumor sizes. Future work will focus on further enhancing the model’s ability to handle complex, heterogeneous data and optimizing its performance across different imaging modalities.

## Figures and Tables

**Table 1:** Comparison of segmentation performance between MSAM, U-Net, nnU-Net, UNETR, and SwinUNETR using different modality combinations.

	Whole Tumor		Tumor Core		Enhancing Tumor	
	DSC	HD95	DSC	HD95	DSC	HD95
U-Net-SD	84.35	5.98	85.64	3.02	81.16	2.39
U-Net-S	84.61	7.02	84.14	3.73	81.20	3.38
U-Net-D	68.01	13.51	52.71	18.81	31.79	17.37
nnU-Net-SD	85.47	5.51	87.21	2.89	82.15	2.21
nnU-Net-S	85.49	5.09	86.94	2.86	82.15	2.39
nnU-Net-D	73.52	9.42	65.90	12.19	46.23	10.55
UNETR-SD	83.83	10.32	81.76	6.61	79.60	4.32
UNETR-S	83.50	9.34	81.18	7.79	79.24	5.86
UNETR-D	63.95	33.20	44.24	43.84	31.06	35.22
SwinUNETR-SD	83.54	8.62	81.72	5.37	79.26	3.68
SwinUNETR-S	83.97	7.16	80.86	5.72	78.90	4.87
SwinUNETR-D	63.49	13.75	40.09	23.68	26.23	21.80
MSAM-SD	89.15	4.49	86.72	3.42	80.83	2.00
MSAM-S	89.27	4.24	87.05	3.34	81.23	2.00
MSAM-D	82.81	4.95	69.95	22.05	54.56	26.13

**Table 2:** Comparison of FFB ablation experimental results.

	Whole Tumor		Tumor Core		Enhancing Tumor	
	DSC	HD95	DSC	HD95	DSC	HD95
MSAM-SD	89.15	4.49	86.72	3.42	80.83	2.00
MSAM-S	89.27	4.24	87.05	3.34	81.23	2.00
MSAM-D	82.81	4.95	69.95	22.05	54.56	26.13
SAM-FLAIR	88.88	4.68	76.56	10.11	58.57	9.14
SAM-T1	85.09	4.76	67.83	23.56	56.15	14.02
SAM-T1gd	82.88	4.76	85.70	7.44	80.13	4.14
SAM-T2	86.62	4.82	76.60	11.20	60.44	15.69
SAM-ad	80.68	5.66	67.06	35.32	48.85	36.36
SAM-fa	79.25	6.13	68.17	36.68	51.75	34.01
SAM-rd	82.01	5.16	64.96	36.40	51.75	25.82
SAM-tr	81.93	5.22	66.55	32.55	52.94	21.33

**Table 3:** Dice Score Results for Different Prediction Modes and Missing Modalities. ∘ denotes a missing modality, • indicates the present modality, and bold type highlights the optimal value for the same prediction mode and with the same missing modality. FTFP: Full Modality Training and Full Modality Prediction, MTFP: Missing Modality Training and Full Modality Prediction, FTMP: Full Modality Training and Missing Modality Prediction, MTMP: Missing Modality Training and Missing Modality Prediction.

	FLAIR	T1	T1gd	T2	FTFP	MTFP	FTMP	MTMP
WT	∘	•	•	•	**89.27**	87.44	79.54	**85.58**
	•	∘	•	•		86.92	**86.92**	86.76
	•	•	∘	•		87.25	86.60	**87.17**
	•	•	•	∘		87.23	86.93	**86.16**
TC	∘	•	•	•	87.05	86.83	85.73	**86.81**
	•	∘	•	•		87.20	86.75	**87.21**
	•	•	∘	•		86.16	73.52	**80.31**
	•	•	•	∘		**87.36**	85.53	**87.20**
ET	∘	•	•	•	81.23	**81.28**	80.49	**81.30**
	•	∘	•	•		80.81	80.79	**80.89**
	•	•	∘	•		79.44	46.42	**64.41**
	•	•	•	∘		80.90	80.62	**80.87**

## Data Availability

The original data presented in the study are openly available in UPenn-GBM at doi.org/10.7937/TCIA.709X-DN49.

## Abbreviations

The following abbreviations are used in this manuscript:

MSAM	Multi-modal Segment Anything Modal
DSC	Dice Score
HD95	95% Hausdorff Distance
FT	Full Modality Training
MT	Missing Modality Training
FP	Full Modality Prediction
MP	Missing Modality Prediction
WT	Whole Tumor
TC	Tumor Core
ET	Enhancing Tumor
